# A new therapeutic approach in Gorham–Stout disease: a case report

**DOI:** 10.3389/fsurg.2023.1225209

**Published:** 2023-09-08

**Authors:** Katarzyna Stawarz, Adam Galazka, Filip Kissin, Jakub Zwolinski

**Affiliations:** Head and Neck Cancer Department, Maria Sklodowska-Curie National Research Institute of Oncology, Warsaw, Poland

**Keywords:** vanishing bone syndrome, osteolytic lesion, rare disease, Gorham–Stout syndrome, chylothorax

## Abstract

**Background:**

Gorham–Stout disease is a rare condition of unknown prevalence and unknown exact cause. Its pathogenesis is based on enhanced osteoclastic activity leading to bone resorption and bone replacement by distended lymphatic vessels. Because of its rarity and a various range of symptoms the disease may give, diagnosis is challenging and a strong index of suspicion is required. Although it is a benign condition, the prognosis may be unpredictable. The treatment options suggested so far are limited, and every case should be provided with the best individual approach. Herein, we present a case report of Gorham–Stout disease managed with a regular lump drainage with a good response and control of the patient symptoms over a period of 20 years.

**Case report:**

A 23-year-old male was admitted to the Head and Neck Cancer Clinic with a 6-month history of a left-sided neck lump. Other symptoms reported were neck pain and general weakness. The basic laboratory tests were within normal limits. On physical examination, a large round lump on the left side of a patient's neck and left armpit were noticed. They were about several centimeters in diameter, soft on palpation, but firmly attached to the underlying tissue. CT scan revealed large lymphatic left-sided masses of the neck and axillary fossa and multiple osteolytic lesions in the patient's vertebrae. Together with the biopsy findings and imaging studies, a diagnosis of Gorham–Stout Syndrome was made. The patient was then scheduled for a regular cystic drainage with good control of a disease for over a period of 20 years.

**Conclusion:**

Gorham–Stout disease is a rare challenging condition, and the available treatment options remain sparse. Although surgical approach is effective, it is not always possible. In addition, the risk of radiotherapy-induced malignancy shows that this therapy may eventually result in unfavorable response. Depending on symptoms and the disease location, this condition requires an individual treatment plan. The presented case illustrates that a minimally invasive approach may result in a good control of the Gorham–Stout syndrome and may stand as an alternative treatment option for some patients with this condition.

## Introduction

Gorham–Stout disease (GSD) is a rare entity with unknown prevalence. According to current publications, there are about 350 cases reported so far (1). Although some theories have been suggested, the exact cause of the disease remains unclear. The pathogenesis of the Gorham–Stout syndrome is probably caused by enhanced osteoclastic activity and uncontrolled proliferation of vascular structures within bones leading to bone destruction and bone replacement by vessels and fibrosis (2). The syndrome mainly affects children or young adults. Early on, patients may be asymptomatic but usually a bone fracture with impaired bone healing and severe localized pain are the first symptoms (3). Other complaints such as body deformity or difficulty breathing occur once the disease progresses and sometimes those symptoms are the reasons that urge patients to see a physician (4). Although benign in origin, this disease may have unpredictable outcomes. The disease progression is hard to assess as it may stabilize for years, go spontaneously into remission, and reoccur after years or may even lead to death at the early onset (5). A high index of suspicion and extensive workup are needed to make a diagnosis of Gorham–Stout disease. CT scan, MRI, or PET scan rather than plain radiographs are the best imaging studies displaying both osteolytic lesions as well as lymphangiomas. Treatment options although sparse may sometimes lead to spontaneous disease arrest after the provided treatment (6). Surgical approach and radiotherapy used to be mostly recommended treatment options although there is a risk of radiotherapy-induced malignancy several years after the treatment (7). Pharmacological therapy is mainly based on vitamin D and calcium agents as well as bisphosphonates. While the former are generally well tolerated, their effectiveness is limited, and the latter may be related to serious side effects such as jaw necrosis (8, 9). Corticosteroids, interferon alpha, cisplatin, or cyclophosphamide among others are medications that may be used in the management of Gorham–Stout syndrome unresponsive to other therapeutic measures (Table 1). Recently, sirolimus, which is a member of macrolide antibiotics, is another agent reported to be effective in some patients with Gorham–Stout syndrome. Its action is based on the inhibition of cell proliferation and angiogenesis through blocking the mTOR signaling pathway. The latest data indicate that it may lead to decreasing the size of lymphangiomas in some patients (10). Other medications such as octreotide, corticosteroids or cisplatin, and cyclophosphamide are reported to be used in patients with GSD but their effectiveness is limited. As such, their use is basically perceived as a last treatment option in patients without any response to other therapies (Table 1). These medications mentioned above may be used alone or in combination with other drugs such as bisphosphonates or radiotherapy (2, 11). Gorham–Stout disease is excluded in diagnosis often and should be always considered when osteolytic lesions are suspected. The laboratory tests are usually normal apart from alkaline phosphatase, which might be elevated. There are some diagnostic criteria that may be used in the diagnosis of Gorham–Stout syndrome—(i) positive biopsy: angiomatous tissue with abnormal lymphatic channels and numerous osteoclasts; (ii) absence of cellular atypia; (iii) minimal or no osteoblastic response and absence of dystrophic calcifications; (iv) evidence of locally progressive bone resorption; (v) non-expansive, non-ulcerative lesion; (vi) absence of visceral involvement; (vii) osteolytic radiographic pattern; and (viii) negative hereditary, metabolic, neoplastic, immunologic, and infectious etiology (12). As it is almost obvious that a majority of physicians will never deal with a vanishing bone syndrome, a multidisciplinary team with a thorough approach is indispensable to confirm the diagnosis. Herein, we present a case report of Gorham–Stout disease managed with regular lump drainage with a good response and control of the patient symptoms over a period of 20 years. Since there is no gold standard treatment for Gorham–Stout disease, it appears that the presented approach may stand for an alternative management plan of this incurable condition.

**Table 1:** Medications used in Gorham–Stout disease

List of medications used in Gorham–Stout disease	
Bisphosphonates	Cyclophosphamide
Calcium	Vincristine
Vitamin D	Sunitinib
Corticosteroids	Taxol
Interferon α-2b	Hydroxychloroquine
Octreotide	Acetazolamide
Bevacizumab	Calcitonin
Propranolol	Sirolimus

## Case report

**Figure 1:**
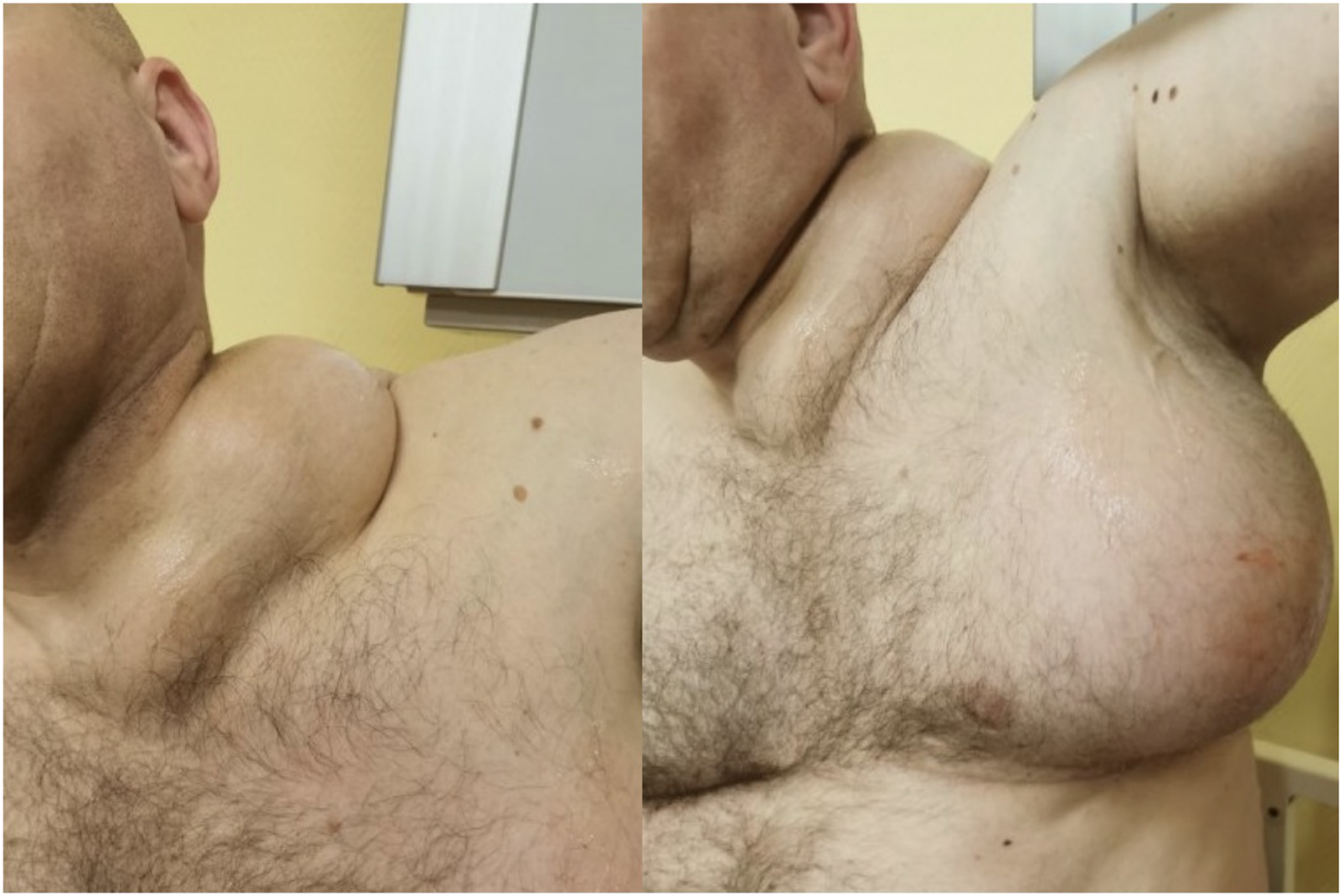
(A) A left side neck lump with unchanged skin. (B) Left armpit lump with unchanged skin.

**Figure 2:**
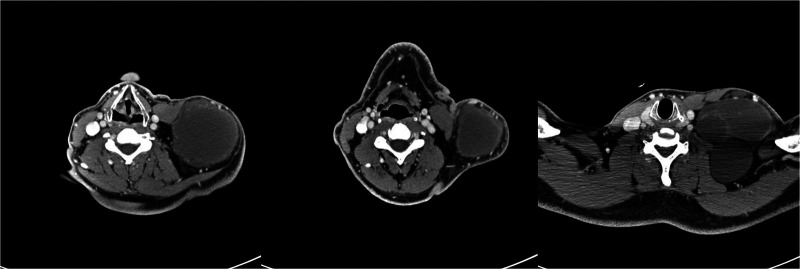
A CT scan of neck mass showing left jugular neck compression.

**Figure 3:**
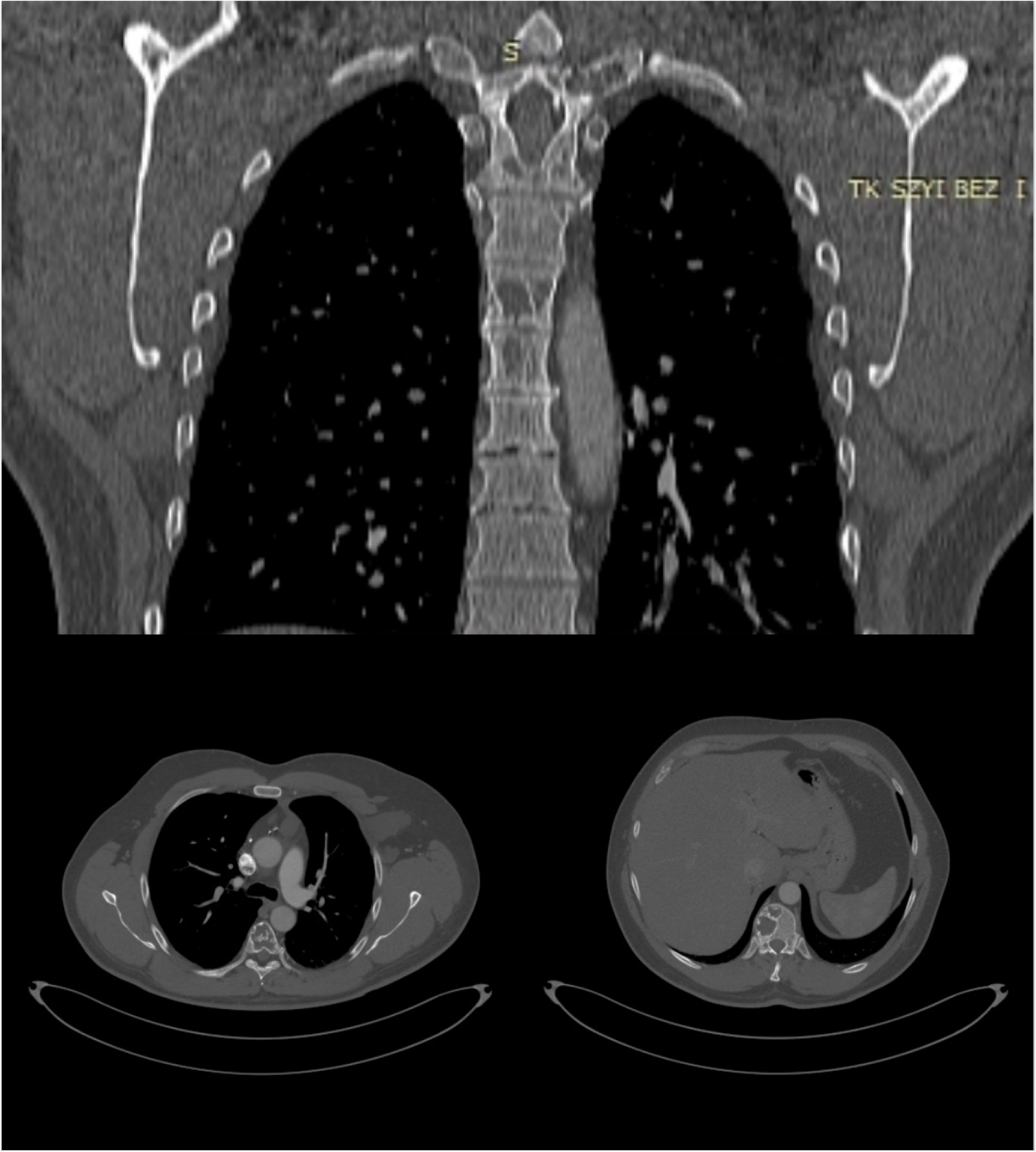
A CT scan of thoracic vertebrae displaying massive osteolytic lesions.

A 23- year-old Caucasian man of Polish origin was seen in The Head and Neck Cancer Clinic of the National Institute of Oncology in Warsaw with a chief compliant of a left-sided neck lump. The patient had noticed the painless mass 6 months earlier. Other symptoms reported were neck pain, which increased in frequency and intensity over time; general weakness; and occasional breathing difficulty. His medical history was insignificant for any surgeries or medical conditions. The patient denied alcohol use disorder, smoking cigarettes, or any recreational drugs. He was employed as a driver. There was no family history of genetic, metabolic, or neoplastic disorders. On presentation, his vital signs were within normal limits with heart rate of 85, respiratory rate of 126/78 mm Hg, and temperature of 36.7°C. On physical examination, a large round lump on the left side of neck above the clavicle was noticed (Figure 1A). It was about 10 cm in diameter, soft on palpation, but firmly attached to the underlying tissue. The skin overlying the mass was normal with no redness or edema. Further investigation revealed a similar mass in the patient's left axillary fossa (Figure 1B). The axillary lump was similar to that one on the neck in its structure, but its dimensions were bigger. Initial laboratory workup for the cystic masses included a complete blood count, C-reactive protein, erythrocyte sedimentation rate, alkaline phosphatase (ALP), calcium, vitamin D level, and a comprehensive metabolic panel that did not reveal any abnormality. Infectious workup that included blood and urine cultures was negative. Moreover, immunological tests checking mainly the rheumatoid factor level were within normal ranges as well. In addition, results of hematological tests together with a bone marrow biopsy were also unremarkable. As the laboratory results usually do not reveal any abnormality in Gorham–Stout disease, other diagnostic measures should be applied. The patient was also screened for neoplasms and hereditary disorders, but genetic testing and tumor markers were negative. Our patient was subsequently scheduled for an ultrasound-guided biopsy and followed by a CT scan. Histopathological report of both masses showed thin-walled, engorged capillary-like vessels, filled with blood cells and fibrin. A computed tomography scan showed a nodular polycyclic fluid lesion with transverse dimensions on the neck of approximately 101 mm × 65 mm, which was spreading from the level of the hyoid bone to the level of the third rib on the left side. This lesion in the upper part of the neck was displaced and compressed the infrahyoid muscles and the sternocleidomastoid muscle. In the middle part of the neck, it compressed the left internal jugular vein and the left subclavian vein. Moreover, it widened the left subclavian space and filled the left armpit (Figure 2). In addition, multiple lytic focal lesions were seen in the vertebral bodies. These lesions had sclerotic margins and several thickened trabeculae (Figure 3). The axillary and neck lumps noticed during physical examination were in fact distended lymphatic vessels creating cystic masses. With reference to the CT scan findings, an open thoracic vertebrae biopsy procedure was performed. Histopathological findings showed resorption of the bone matrix and angiomatous tissue with abnormal lymphatic channels. No cellular atypia was found. There were no calcifications and the osteoblastic response was minimal. Following this, our patient was consulted by a hematologist and an orthopedist to rule out multiple myeloma, lymphangiomatosis, and other skeletal or hematologic disorders. Since the diagnosis of Gorham–Stout disease is challenging, we applied the diagnostic criteria proposed by Heffez et al. On the basis of the clinical presentation, imaging studies, and histopathological findings together with the criteria used, we made a diagnosis of Gorham–Stout disease. The presented findings are consistent with a recurrent cystic lymphangioma as a main disease manifestation as the patient did not complain of spine symptoms. Due to the large size of a mass with both cervical and thoracic vertebrae involvement, the surgical treatment aiming to remove the mass was not possible. Taking into consideration the patient's young age and the risk of secondary malignancy, he did not agree to radiotherapy. He was given vitamin D and calcium and was started on a bisphosphonate regimen. Due to the side effects of bisphosphonates such as diarrhea, flu-like symptoms, and bone pain, the patient was reluctant to proceed with this therapy. While there is no effective treatment that is recommended, the patient was finally offered an approach based on axillary and neck lump drainage to relieve his symptoms and decrease body deformity. The procedure was performed under local anesthesia with the use of 2% lidocaine solution and with ultrasound imaging. The 20 G spinal needle was used for the procedure. Before needle insertion, the site was cleaned and disinfected with an antiseptic agent. The needle location was confirmed with ultrasound imaging, and the drained fluid was sent for a cytological examination. Then, neomycin was put on the site of needle insertion and a dressing was made, which was removed on a follow-up visit 2 days later. The patient’s symptoms were successfully managed through the years from 2003 till 2023. The procedure of drainage was conducted every 6 months as that was the period of time when the fluid accumulated and caused the patient’s condition to worsen. Right after the procedure, the patient did not suffer from any complications related to the biopsy itself nor present any signs of infection. Several years later, our patient was commenced on treatment with sirolimus as it is proven to decrease the volume of lymphatic vessels in some patients, but unfortunately, no disease stabilization was achieved. The follow-up plan included repeated MRI imaging every 1–2 years, which eventually revealed a decrease in the size of patient's neck and axillary cystic masses. Laboratory tests including full blood count together with ALP, calcium, and vitamin D were ordered once per year, but no significant changes have been detected so far. Nevertheless, the patents’ symptoms were well controlled for over 20 years with no significant side effects related to the treatment provided. Moreover, the patient seemed to be confident with the presented approach as the regular visits enabled him to stay active and have the disease under control.

## Discussion

Gorham–Stout disease is a rare condition affecting mostly young adults or children (13). There is no predilection for any sex although some authors report that the disease occurs more commonly in males (14). The general pathogenesis of this syndrome includes enhanced osteoclastic activity leading to bone resorption and proliferation of lymphatic vessels within the invaded bone. This manifests with bone deformity and swelling of the affected region. The upper extremity bones and the maxillofacial region are the most frequently reported disease locations. Symptoms presented by patients may differ but pain caused by a pathological fracture is usually the compliant that makes them seek medical care. This is a condition with an unpredictable outcome and the complications of the disease may be fatal. Pleural effusion or chylothorax caused by lymphangiectasia of vertebral bodies spreading to the chest cavity may lead to a respiratory distress and eventually death. Moreover, the involvement of vertebral bodies can cause spinal cord compression resulting in paraplegia (15). In the presented case scenario, our patient did not demonstrate symptoms caused by spinal involvement. Instead, the patient’s main concern was recurrent cystic lymphangioma, which is a spectrum of GSD (16). In terms of prognosis of patients with Gorham–Stout disease, lymphangiectasia and spinal involvement are considered to be poor prognostic factors (17). Gorham–Stout disease is excluded in diagnosis often, and although some diagnostic criteria have been proposed, a strong index of suspicion is needed to confirm the diagnosis. Imaging studies, histopathological reports, and thorough clinical presentation are needed to diagnose GSD after ruling out inflammatory, genetic, or cancerous origin of a disease (18). The available treatment options are limited and depend on the disease location. Surgical treatment when possible and/or radiotherapy are those steps that used to be recommended. Nevertheless, today there are pharmacological measures that are considered to be main strategies for the management of GSD. Recently, sirolimus as another treatment option proved to be effective in some cases but unfortunately did not result in any symptoms improvement in our patient. Moreover, it has been suggested that taking bisphosphonates may be of great benefit for patients with Gorham–Stout disease (19). The severe side effects as well as the risk of jaw necrosis often make the patients reluctant to take those drugs. Other medications such as calcium and vitamin D although well tolerated do not influence disease progression. Still, as the age of patient admission and general complaints differ, the choice of the best treatment plan remains challenging. Surgical procedure with following reconstruction seems to be most effective. As radiotherapy may lead to malignancy several years after treatment, it is not always a good therapeutic option especially for young patients. In the presented case scenario, a decision of supportive care aiming to symptom relief with the minimally invasive procedure was the most accurate approach to our beliefs. The patient was first presented to the clinic at the age of 23 and surgical intervention early on that time was not yet possible to perform. Due to the patient's young age and the significant risk of radiotherapy-induced malignancy, the decision of radiotherapy that might be effective was postponed. In addition, assessment of the patient revealed a lack of response to the pharmacological treatment provided. Moreover, regarding the fact that the diagnosis was made more than 20 years ago, the treatment options reported in the literature at that time were sparse. However, over time, even with new possible therapies suggested, the presented treatment plan remained to be effective. The patient was seen every 6 months with good disease control and no negative side effects caused by our treatment. The biggest limitation of this case study though is the inability to generalize the presented findings to other, similar cases. A larger group of patients diagnosed with Gorham–Stout syndrome with the therapeutic approach suggested in this case study is needed to properly evaluate this method as a new therapeutic option. Nevertheless, this case report stands for a valuable and informative method of investigation, providing in-depth insights and understanding of this rare entity and a possible new therapy. In conclusion, this case report proves that a minimally invasive approach based on a regular fluid drainage may be effective in the management of some patients suffering from Gorham–Stout syndrome.

## Data Availability

The original contributions presented in the study are included in the article/Supplementary Material, further inquiries can be directed to the corresponding author.

## References

[1] Angelini A Mosele N Pagliarini E Ruggieri P. Current concepts from diagnosis to management in Gorham-Stout disease: a systematic narrative review of about 350 cases. EFORT Open Rev. (2022) 7(1):35–48. 10.1530/EOR-21-0083 35076412 PMC8788153

[2] Bosco F Giustra F Faccenda C Boffano M Ratto N Piana R. Gorham-Stout disease: a rare bone disorder. J Orthop Rep. (2022) 1(2):100028. 10.1016/j.jorep.2022.04.005

[3] De Keyser CE Saltzherr MS Bos EM Zillikens MC. A large skull defect due to Gorham-Stout disease: case report and literature review on pathogenesis, diagnosis, and treatment. Front Endocrinol. (2020) 11:37. 10.3389/fendo.2020.00037 PMC7012895 32117063

[4] Vaishya R Vaish A Singh LK Baweja P. Management of a pathological fracture in a rare case of Gorham Stout disease of the hip with a mega prosthesis. J Orthop. (2020) 18:177–80. 10.1016/j.jor.2019.08.003 32042222 PMC7000433

[5] Heyd R Micke O Surholt C Berger B Martini C Füller J Radiation therapy for Gorham-Stout syndrome: results of a national patterns-of-care study and literature review. Int J Radiat Oncol Biol Phys. (2011) 81(3):e179–85. 10.1016/j.ijrobp.2011.01.006 21345608

[6] Ruggieri P Montalti M Angelini A Alberghini M Mercuri M. Gorham-Stout disease: the experience of the Rizzoli Institute and review of the literature. Skeletal Radiol. (2011) 40(11):1391–7. 10.1007/s00256-010-1051-9 20972870

[7] Khanna L Prasad SR Yedururi S Parameswaran AM Marcal LP Sandrasegaran K Second malignancies after radiation therapy: update on pathogenesis and cross-sectional imaging findings. RadioGraphics. (2021) 41(3):876–94. 10.1148/rg.2021200171 33891523

[8] Hu P Yuan XG Hu XY Shen FR Wang JA. Gorham-Stout syndrome in mainland China: a case series of 67 patients and review of the literature. J Zhejiang Univ Sci B. (2013) 14:729–35. 10.1631/jzus.B1200308 23897792 PMC3735973

[9] Maillot C Cloche T Le Huec JC. Thoracic osteotomy for Gorham-Stout disease of the spine: a case report and literature review. Eur Spine J. (2018) 27(9):2285–90. 10.1007/s00586-014-3613-3 25331037

[10] Liang Y Tian R Wang J Shan Y Gao H Xie C Gorham-Stout disease successfully treated with sirolimus (rapamycin): a case report and review of the literature. BMC Musculoskelet Disord. (2020) 21:577. 10.1186/s12891-020-03540-7 32843029 PMC7446191

[11] Brodszki N Länsberg JK Dictor M Gyllstedt E Ewers SB Larsson MK A novel treatment approach for paediatric Gorham-Stout syndrome with chylothorax. Acta Paediatr. (2011) 100:1448–53. 10.1111/j.1651-2227.2011.02361.x 21605166

[12] Heffez L Doku HC Carter BL Feeney JE. Perspectives on massive osteolysis. Report of a case and review of the literature. Oral Surg Oral Med Oral Pathol. (1983) 55:331–43. 10.1016/0030-4220(83)90185-8 6574409

[13] Rana I Buonuomo PS Mastrogiorgio G Del Fattore A Jenkner A Barbuti D Expanding the spectrum of Gorham Stout disease exploring a single center pediatric case series. Lymphology. (2021) 54(4):182–94. PMID: . 35073622

[14] Momanu A Caba L Gorduza NC Arhire OE Popa AD Ianole V Gorham-Stout disease with multiple bone involvement—challenging diagnosis of a rare disease and literature review. Medicina. (2021) 57:681. 10.3390/medicina57070681 34356962 PMC8304881

[15] Xing Q Miao M Zhang Q Wu Y He F. Gorham-Stout disease affecting the spine with cerebrospinal fluid leakage and Chiari-like tonsillar herniation: a rare case report and review of literature. BMC Neurol. (2023) 23(59). 10.1186/s12883-023-03092-y PMC9896703 36737721

[16] Johnstun J Brady L Simstein R Duker N. Chronic recurrent Gorham-Stout syndrome with cutaneous involvement. Rare Tumors. (2010) 2(3):e40. 10.4081/rt.2010.e40 21139956 PMC2994520

[17] Patel DV. Gorham's disease or massive osteolysis. Clin Med Res. (2005) 3:65–74. 10.3121/cmr.3.2.65 16012123 PMC1183435

[18] Zhang L Li J Yao F Chen Y Zhang S Lv H Treatment of Gorham-Stout disease with bisphosphonates and total hip arthroplasty: a case report. Front Surg. (2023) 10:1078869. 10.3389/fsurg.2023.1078869 36793315 PMC9922857

[19] Schneider KN Masthoff M Gosheger G Klingebiel S Schorn D Röder J Gorham-Stout disease: good results of bisphosphonate treatment in 6 of 7 patients. Acta Orthop. (2020) 91(2):209–14. 10.1080/17453674.2019.1709716 31928107 PMC7144312

